# Supplementary material for: Probiotics-Containing Yogurt Ingestion and H. pylori Eradication Can Restore Fecal Faecalibacterium prausnitzii Dysbiosis in H. pylori-Infected Children
Source: Biomedicines. 2020 Jun 1;8(6):146. doi: 10.3390/biomedicines8060146 (PMC7344718; doi:10.3390/biomedicines8060146)
Supplement: Supplementary file 1 [file biomedicines-08-00146-s001.zip › Supplement files/Supplement Table S1.docx]

**Supplement Table 1.** Comparisons of fecal calprotectin, lactoferrin, IL-6, TGF-β1, and sIgA levels between *H. pylori*-infected and non-infected children

| Parameters\groups | *H. pylori*-infected  (n=22) | Controls  (n=10) | *p* value |
| --- | --- | --- | --- |
| Calprotectin (μg/g) | 13.2±21.5 | 2.5±2.3 | 0.13 |
| Lactoferrin (μg/g) | 15.4±25.1 | 6.8±9.7 | 0.18 |
| IL-6 (pg/ml) | 3.4±8.8 | 1.3±0.4 | 0.27 |
| TGF-β1 (ng/ml) | 12.0±7.7 | 7.0±3.3 | 0.02 |
| sIgA (μg/ml) | 240.3±270.0 | 505.0±377.2 | 0.07 |

TGF, transforming growth factor; sIgA, secretory immunoglobulin A.
