# Supplementary figures and images for: Probiotics-Containing Yogurt Ingestion and H. pylori Eradication Can Restore Fecal Faecalibacterium prausnitzii Dysbiosis in H. pylori-Infected Children
Source: Biomedicines. 2020 Jun 1;8(6):146. doi: 10.3390/biomedicines8060146 (PMC7344718; doi:10.3390/biomedicines8060146)

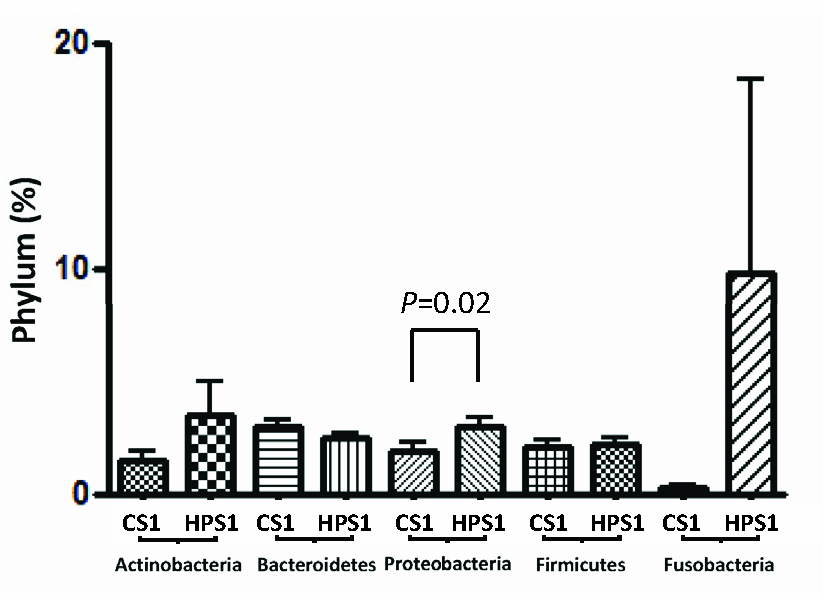

Supplement: Supplementary file 1 [file biomedicines-08-00146-s001.zip › Supplement files/Supplement Figure S2.tiff]

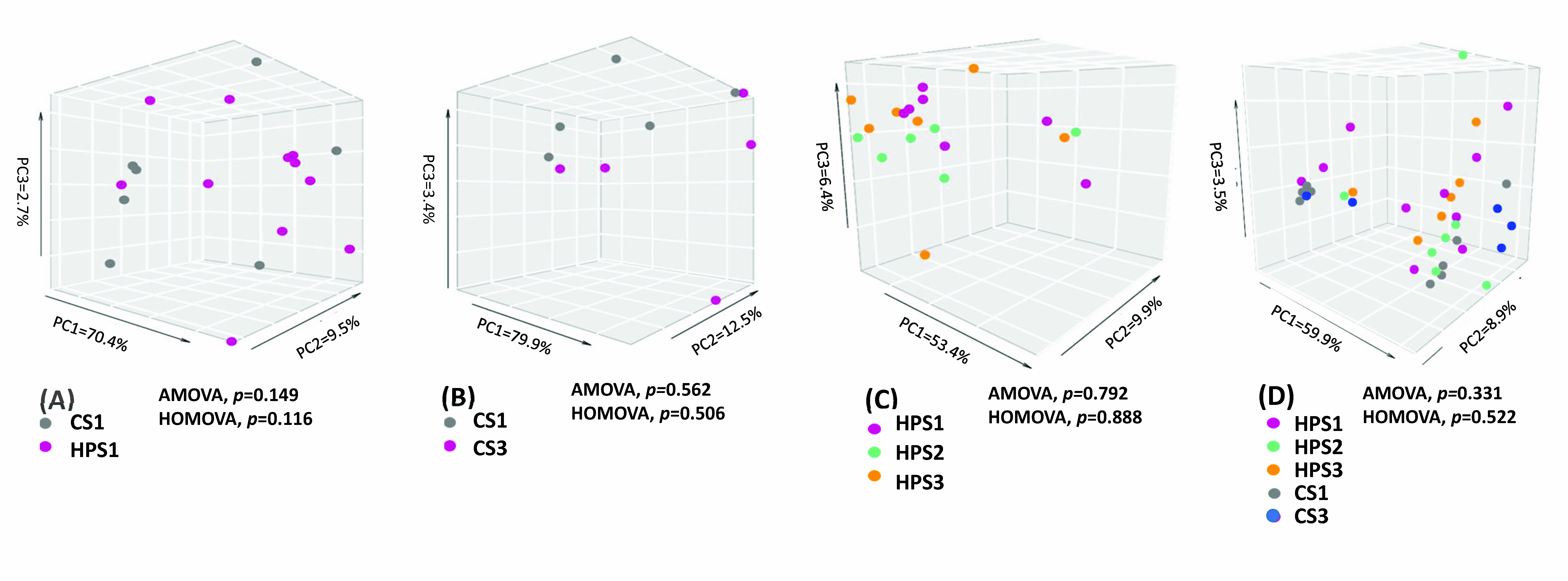

Supplement: Supplementary file 1 [file biomedicines-08-00146-s001.zip › Supplement files/Supplementary Figure S1-rv.tiff]

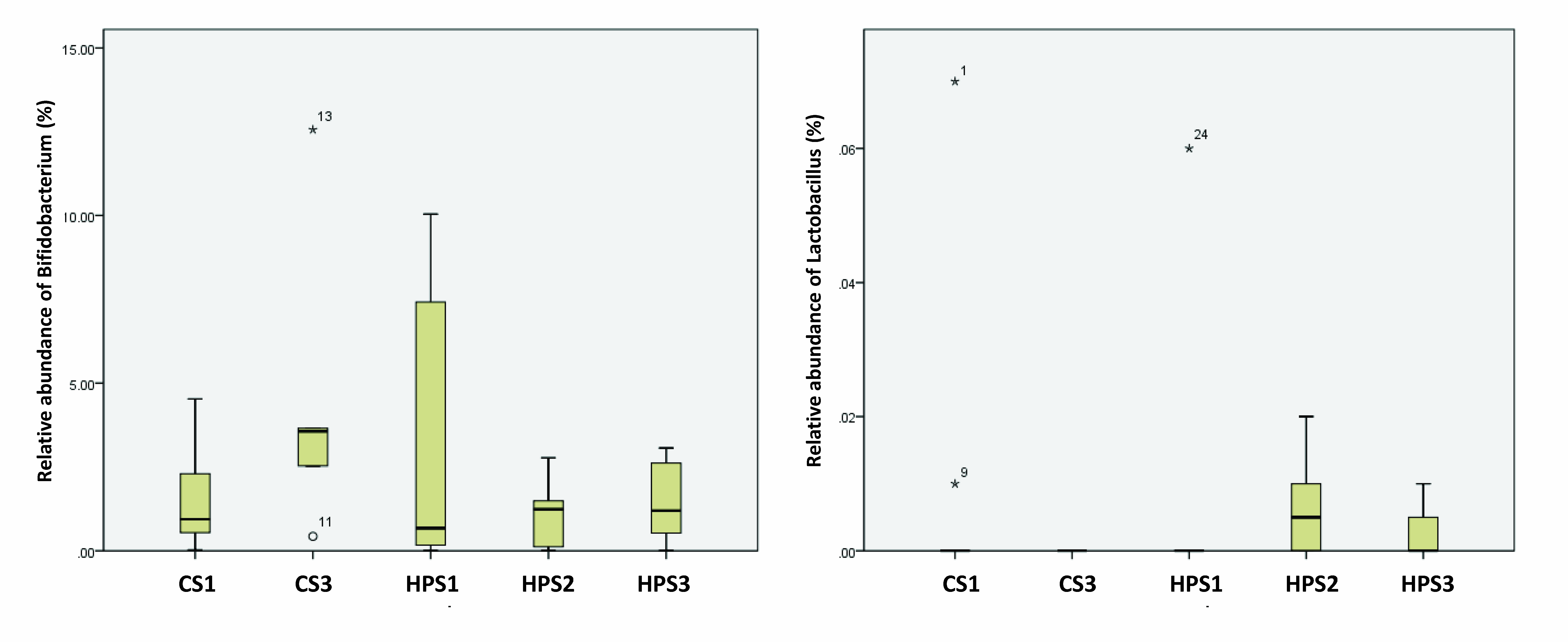

Supplement: Supplementary file 1 [file biomedicines-08-00146-s001.zip › Supplement files/Supplementary Figure S3.tiff]
